# Experimental Study of a Heat Pump for Simultaneous Cooling and Desalination by Membrane Distillation

**DOI:** 10.3390/membranes11100725

**Published:** 2021-09-23

**Authors:** Ahmadou Tidiane Diaby, Paul Byrne, Patrick Loulergue, Ousmane Sow, Thierry Maré

**Affiliations:** 1 Laboratoire du froid, des systèmes énergétiques et thermiques (Lafset), Cnam—Hesam Université, 292 rue Saint Martin, 75003 Paris, France; ahmadou-tidiane.diaby@lecnam.net; 2 Laboratoire de Génie Civil et de Génie Mécanique, Université de Rennes, F-35000 Rennes, France; paul.byrne@univ-rennes1.fr (P.B.); thierry.mare@univ-rennes1.fr (T.M.); 3 Univ Rennes, CNRS, ISCR–UMR 6226, F-35000 Rennes, France; 4 Laboratoire Eau, Energie, Environnement et Procédés Industriels—Ecole Supérieure Polytechnique (ESP)-Université Cheikh Anta Diop, Dakar 10700, Senegal; sowmane@yahoo.fr

**Keywords:** simultaneous demand, heating, cooling, desalination, membrane distillation

## Abstract

Heat pump systems can simultaneously produce cooling energy for space cooling in hotels, office and residential buildings and heat for desalination using membrane distillation (MD). The MD technique uses a heat input at a temperature compatible with the levels of heat pump condensers (<60 °C). A heat pump prototype coupled with an air-gap membrane distillation unit was constructed and tested. This paper presents the experimental study on a lab-scale prototype and details the two operating modes “continuous” and “controlled” simulating an air conditioning system and a food storage, respectively. The experimental results enable to analyze the performance of the prototype and the physical phenomena involved. Finally, the study shows that this system could be a promising solution to help supplying freshwater to people in hot regions of the world.

## 1. Introduction

The global cooling demand increases due to climate change and to the growing population in developing countries [1,2]. Cooling systems generally reject lost heat to the ambient air, contributing to the heat island effect. The heat island effect is the accumulation of heat in dense city centers. The superposition of condensers on the facades of buildings is a non-sense on a thermal point of view. They heat the air near to the condensers above inducing a lower performance.

Climate change also affects water resources worldwide. Globally, arid regions become more arid [3]. Seawater desalination has been developed as an alternative to the classical treatment of surface and ground waters to enlarge the potential resources for freshwater production. Therefore, desalination is used to provide freshwater in regions of the world suffering from water scarcity. The cumulative capacity of desalination systems by 18,983 plants installed worldwide is a daily production of 92.5 million cubic meters per day at the end of 2018 [4].

The objective of our work is to couple the condenser of a vapor compression cooling system to a membrane distillation unit. Such a hybrid system could be used to valorize the heat lost by buildings or to equip standalone devices and can be named a heat pump for simultaneous cooling and desalination. The following literature review focusses on the subjects of membrane distillation, of the associated heat sources and of heat pumps dedicated to desalination.

### 1.1. Principles of membrane distillation

Membrane distillation (MD) is a low area footprint membrane-based thermal separation technique. A hydrophobic and porous membrane (pore diameter typically in the range 0.1–0.5 µm) is used to separate two compartments: the feed channel containing the fluid, to be treated, and the permeate channel receiving the molecules able to cross the membrane. Inorganic salts are nonvolatile components in contrast to water, which is volatile. A temperature difference is maintained between the two compartments in order to create a vapor pressure difference (acting as the driving force) so that a fraction of the liquid feed solution can be vaporized and transfer through the membrane. On the other hand, the hydrophobic nature of the membrane prevents the passage of liquid water and non-volatile solutes through the membrane thus allowing the recovery of high purity permeate theoretically cleared of nonvolatile components [5]. Furthermore, one of the major interests of this technique is the low sensibility towards feed solution salinity thus allowing to concentrate the solutions up to their saturation point without any important flux decline. For this reason, MD appears as a good candidate for water desalination including freshwater production from seawater [6,7,8,9], wastewater treatment [10,11,12], and brine treatment [7,13,14] but also for the separation of volatile molecules contained in salt-containing aqueous complex mixtures [15,16].

Different MD techniques exists depending on the membrane module configuration. The advantages and drawbacks of the main configuration are presented in Table 1. In the application of a standalone system, the robustness, the simplicity, and the reliability of the equipment has to be maximum. The higher wettability of DCMD and PGMD is a major drawback that has to be avoided for such application. In spite of the lower performance of the system, the AGMD technique was chosen for the prototype because of its simplicity.

### 1.2. Membrane Distillation and Heat Sources

The MD separation process requires a temperature difference between the two sides of the membrane that can be created using low-grade thermal energy. MD application typically requires a hot feed solution temperature in the range of 40–80 °C while the temperature of the permeate side is kept lower [17]. Therefore, this unique feature makes MD an excellent candidate for a coupling with renewable energy or waste heat recovery. A number of low-grade heat sources (T < 130 °C) have been considered for MD including solar energy, geothermal energy, and waste heat from industrial or urban facilities.

#### 1.2.1. Solar Energy

Solar energy can be exploited either by photovoltaic systems (conversion of sunlight into electricity) or by thermal harvesting devices (conversion of sunlight into heat). Due to its thermal-driven nature, MD is suitable for direct coupling with thermal solar energy. In particular, this process can be of great interest in arid regions having abundant solar resources and access to seawater such as Middle East, Southeast Asia, Australia, or the Mediterranean basin.

Numerous studies have been carried out to couple MD with different types of solar energy harvesting devices including flat-plate collectors, vacuum-tube collectors, compound parabolic collectors, solar stills, and salinity-gradient solar ponds [18,19]. These studies have been carried out both at lab-scale and demonstration-scale demonstrating the technical interest of the coupling between solar energy and MD [20]. In particular, solar-MD could have a promising application as a standalone off-grid device for the production of fresh water in remote areas (coupling both thermal and photovoltaic solar systems with MD). To date, the economic competitiveness of solar-MD is still under debate [21] because MD is in its growth phase [20] (only few commercial membrane modules are available [22]) and because the cost of thermal solar energy (including land cost for solar system implementation [23]) is still large. However, solar-MD competitiveness should benefit from the on-going efforts to develop energy efficient MD systems, from the decrease of the cost of membrane modules associated with the commercial development of MD units and from the feedback of the exploitation of the growing number of pilot and commercial facilities. Development of an alternative heating method based on the direct heating of feed water at the membrane surface using photothermal heating is also considered [24]. However, such technology has mostly been tested at lab/pilot-scale.

#### 1.2.2. Geothermal Energy

Geothermal energy is the source of heat produced and stored in the Earth [25]. Geothermal systems, i.e., “convecting water in the upper crust of the Earth” [26], can be of two types: liquid-dominated systems or vapor-dominated systems. Accordingly, wide variations of temperature can be observed from one system to the other ranging from the temperature of the Earth’s surface to more than 225 °C. Depending on the temperature of the geothermal resource, different uses of the energy have been envisaged including bathing and swimming, aquaculture, greenhouse and space heating, geothermal heat pumps, or electricity production [27]. The low-enthalpy geothermal resources (<125 °C) are particularly interesting in view of coupling with MD.

The main advantage related to the use of geothermal energy for MD systems is that this source of energy is perpetual, allowing a constant heat supply to the process [28,29]. Furthermore, geothermal energy can be found in many countries and on all continents. Additionally, another advantage to the use of geothermal energy in MD is that the fluid to be treated (water coming from the Earth) is also the one transporting the energy required to run the process (heat coming from the Earth) thus avoiding the necessity of heat storage or heat transfer [30]. However, the use of geothermal energy is associated with the necessity of drilling, which might lead to high capital costs [23], and could be spatially limited to the area of geothermal wells.

#### 1.2.3. Waste Heat

Large amounts of heat are wasted around the world in the industrial, the transportation, the (residential/commercial) building and the electricity generation sectors.

Industrial low-grade waste heat can typically be defined as the heat that cannot be viably recovered within a process [31]. Despite economically- and environmentally-driven efforts made by the different industrial sectors to optimize energy consumption, high amounts of energy are still lost under the form of heat. Therefore, Forman et al. [32], recently estimated that 72% of the world global primary energy (estimated to be 474,171.10^15^ J in 2012) is lost after conversion while Cullen and Allwood [33] estimated that only 11% of the primary energy (estimated to be 474.10^18^ J in 2005) is converted into useful energy. Obviously, the calculation of the global waste heat potential in the world, one country or one industrial sector cannot lead to an exact value due to the necessity to estimate and collect a number of parameters from the field or through theoretical approaches. However, it clearly appears that a large amount of energy is wasted and that it exists substantial space to improve energy management, in particular through low-grade heat valorization. Forman et al. have shown that a number of sectors reject heat in temperature ranges compatible with the coupling with MD [32]. According to their theoretical calculations, roughly 63% of the heat rejected (245,717.10^15^ J in 2012) is at a temperature below 100 °C. The electricity generation sector accounts for about 60% of the total waste heat with temperature < 100 °C followed by the transportation sector (18%), residential/commercial (mainly buildings) sector (13%), and the industrial sector (9%).

A number of studies has been carried out to valorize this waste heat for MD application. In particular, the most promising application of waste heat-driven MD is in the field of electricity generation, in good agreement with the fact that this sector is the main producer of waste heat streams having a temperature compatible with MD. As reviewed by Thomas et al., several commercial or demonstration-scale MD facilities fed with waste heat from power plants have been implemented in the last 10–15 years [20]. However, one should keep in mind that the development of such facilities is spatially limited by the locations of the power plants. The same drawback is observed when considering the recovery of waste-heat from the industrial sector. On the contrary, waste heat from the transportation sector can be found in a large number of locations. However, due to the diffuse nature of this energy deposit, it is, in practice, extremely complex to valorize such waste heat streams for MD application. To the best of our knowledge, the only published studies are related to the application of MD on board ships [34,35].

Another important source of waste heat might be the data center industry. In 2010, data centers were responsible for roughly 1.5% of the world electricity consumption with an important annual growth as high as 15–20% [36]. Despite the fact that considerable effort has been made to optimize the energy performance of data centers, the energy consumption of data centers has increased by 32% between 2005 and 2018. This energy consumption seems likely to grow even more in the next couple of years [37] supported by new trends including video streaming, social media, big data, bitcoin, artificial intelligence, and digitalization of business, processes, and production flows leading to more and more data being stored and processed in data centers [38]. A large amount of this electric energy is actually converted into heat. The proper operation of the servers of data centers might require dissipating up to 46 kW of heat per square meter of server racks [39]. Typically, three main technologies exist to cool data centers: air-cooling systems, water-cooling systems, or two-phases cooling systems. Depending of the cooling technology, the flux of waste heat might have different temperatures [40,41]. For air–cooled systems, the temperature of heat extracted is typically in the range of 35–45 °C while it is in the range of 50–70 °C for water-cooled data centers. Conversely, two-phase cooling systems can produce waste heat streams having a temperature up to 70–80 °C. A review from Ebrahimi et al. [39] discussed the potential applications to valorize this waste heat. Among others, the production of desalinated water is envisaged using multiple effect distillation. However, such applications have not yet been tested. One major limitation is that multiple effect distillation requires relatively high waste heat temperature around 70 °C [41] thus limiting the possible coupling only to two-phase cooling or requiring the use of cascade vapor compression systems or CO_2_ transcritical systems to reach the higher temperature. On the other hand, MD requires lower temperature and the coupling with all kinds of data centers, cooling systems can be envisaged.

### 1.3. Heat Pumps for Desalination

The operating principle of the heat pump is based on a vapor compression thermodynamic cycle [42]. Heat is recovered from a heat source to evaporate a refrigerant. The refrigerant is compressed to a high-pressure level. Then it changes phase at a higher temperature in the condenser and the heat is rejected to a heat sink. The heat pump can simultaneously produce heating energy at the condenser and cooling energy at the evaporator [43].

Only few heat pumps were already used in desalination because only few desalination methods are suitable to the temperatures of standard heat pumps or air conditioners. Nevertheless, Tan et al. studied a thermoelectric heat pump for simultaneous water treatment and space cooling [44]. The desalination unit uses a sweeping-gas membrane distillation method. The unit has a power consumption of less than 300 W and produces nearly 120 mL per kWh, corresponding to a specific energy consumption of 8.33 kWh/m^3^.

The crystallization method carries out simultaneous cooling, desalination, and heating [3]. The ice formed at the evaporator by freezing seawater is pure. It can be subsequently scraped and melted to produce freshwater. Crystallization has a relatively low energy consumption (9.37 kWh/m^3^ in the publication of Johnson [45]).

Slesarenko presents a heat pump integrated in a desalination process for waste heat recovery and reduced power consumption [46]. However, the heat pump is not the core of the system and does not carry out cooling.

Recently, the potential interest of coupling MD with a heat pump for simultaneous cooling of residential and commercial buildings and clean water production was demonstrated [9,47]. It is estimated that 17% of the global electric energy is consumed by the four billion air conditioning and refrigeration devices installed worldwide [3]. According to their operation, such devices lead to the rejection of high amounts of waste heat outside of the buildings. One major interest of this approach is the co-location of the freshwater and cooling needs (i.e., at building scale). Based on a modelling work and an experimental validation using separated AGMD and heat pump devices, Byrne and co-workers demonstrated the potential interest for such coupling. In a follow-up of this study a prototype unit allowing to demonstrate the interest of coupling MD with heat pumps. The performance of this unit has been evaluated and is presented in this article.

## 2. Materials and Methods: Heat Pump and AGMD

### 2.1. Heat Pump

**Figure 1:**
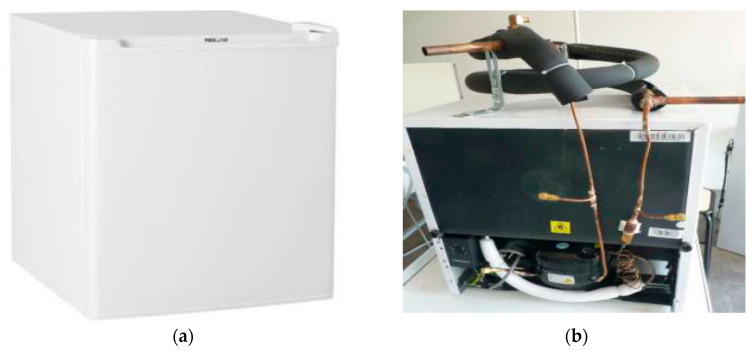
Heat pump for the simultaneous production of cold and heat (**a**) picture of the refrigerator and (**b**) photo of the refrigeration machine modified.

In this experimental study, a refrigerating machine for domestic use constitutes part of the test bench (Figure 1a). It is designed to operate in an ambient temperature between 16 °C and 38 °C. The unit does not function properly outside of this temperature range. If the device is exposed to a too high temperature for a long time, the temperature in the refrigerator will rise above 4 °C. Temperature and refrigerator cooling rate depend on the location, the door opening frequency and the ambient temperature of the room where the appliance is located.

To become a heat pump for simultaneous cooling and desalination, the small refrigerator has been modified (Figure 1b) in order to make a coupling with a membrane distillation cell. The original static condenser was replaced by a tube-in-tube coaxial exchanger to transfer the condensation heat to a water loop. Three pressure taps (shrader valves) at the suction, compressor discharge, and condenser outlet were installed on the refrigeration circuit. The electrical power indicated on the nameplate of the device is 60 W. The refrigerant used is isobutane (R600a) which is a natural fluid with low environmental impact but highly flammable. The high flammability is acceptable for this device because of the refrigerant charge (mass) under the regulatory limit. Given the performance of R290 in simulation for air conditioning and freshwater production [9], the choice of a hydrocarbon seemed relevant for the experimental study. Isobutane happens to be the cheapest refrigerant on the market. However, the thermodynamic properties of isobutane and propane are slightly different.

### 2.2. Air Gap Membrane Distillation Cell

**Figure 2:**
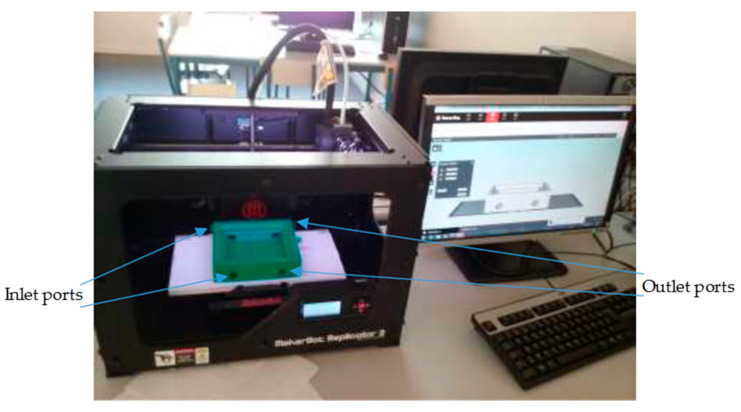
Image of 3D printer and fabricated cold compartment.

An AGMD cell was specifically designed and fabricated to build the prototype unit. The main parameters that determine heat and mass transfer in the module can be varied by adjusting the thickness of the air channel and the flow rate. The volume of the hot channel is reduced to minimize thermal gradient losses because of temperature polarization. The MD module is primarily composed of compartments to accommodate hot feed water and coolant flow channels. The first compartment corresponds to the hot channel and the second, to the cold channel. On each compartment, two inlet holes and two 8 mm diameter outlet holes are located face to face to increase the homogeneity of the flow in the channels and avoid dead-zones that could have occurred if only one entry and one exit existed. This MD cell allows counter-current or co-current flow. After drawing the various components with the SolidWorks software, the unit was 3D printed with a biopolymer (Figure 2).

**Figure 3:**
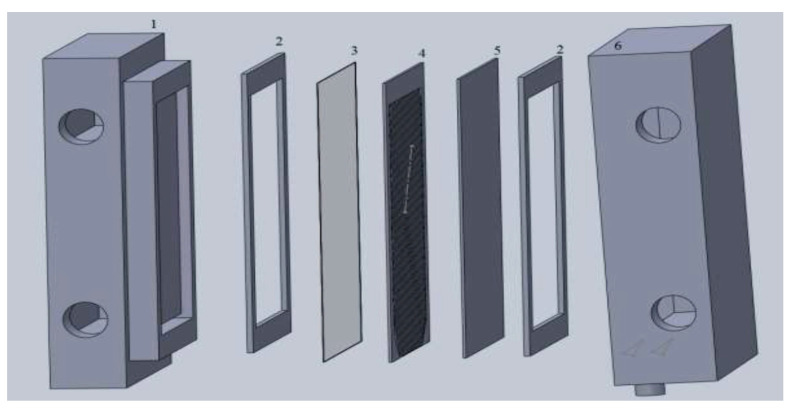
Exploded view of the AGMD module assembly: (1) hot compartment, (2) rubber gaskets, (3) membrane, (4) spacer, (5) condensation plate and (6) cold compartment.

An isometric view of the AGMD cell is shown in Figure 3. This AGMD module has an air gap (air layer) whose thickness is adjustable defined by the thickness of the spacer crossed filaments which separates the two compartments of the cell. The hot feed channel is in direct contact with the membrane. The condensing plate separates the air gap and the cooling channel. Hot and cold channels have front dimensions of 142 mm by 142 mm and thicknesses of 26 mm and 35 mm, respectively. In the hot channel, water passes through a 3.9 mm hydraulic diameter channel. The air gap contains a drain and an outlet tube in the bottom part to evacuate the distillate. The effective permeation surface area is 6.4 × 10^−3^ m². The spacer also serves as a support for the membrane. Rubber gaskets are inserted between the compartments and the metal plate and membrane to provide a seal and prevent damage to the membrane material on the sharp edges of the module when bolts apply pressure. The useful part of the hot compartment and all other elements can be housed in the hollow area of the cold compartment.

**Figure 4:**
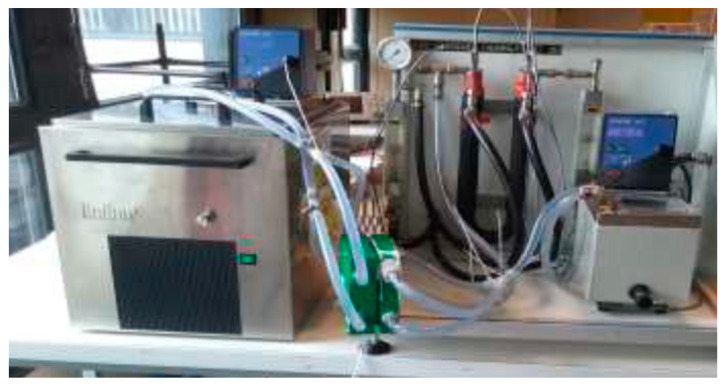
Test bench for the preliminary test.

**Figure 5:**
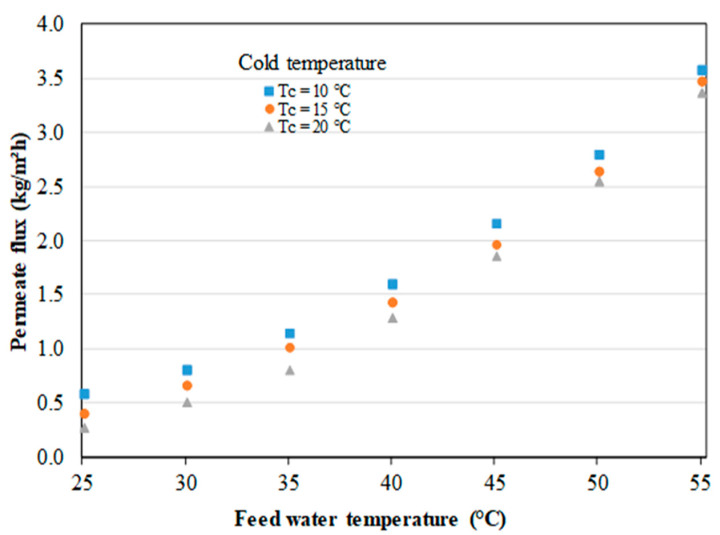
Effect of hot and cold feed water temperatures on permeate flux.

A preliminary study was carried out on the AGMD module. A test bench composed of an AGMD cell, a hot circuit and a cold circuit was designed and finely instrumented (Figure 4). Table 2 gives an overview of the measurement methods applied and the sensors used. Type K thermocouples, volume meters, an electric energy counter were used. A 0.01 g precision scale was also connected to the Labview(National Instruments, Austin, TX, USA) data acquisition system which records the mass of permeate collected continuously. The propylene membrane has a 0.25 µm thickness, a 55% porosity, and a 0.064 μm pore size. The tests were carried out with a cold flow of 1 L/min, a hot flow of 0.6 L/min, and an air-gap thickness of 1.04 mm. The effect of coolant temperature on permeate flux is less important than the effect of hot feed water temperature, as expected [48]. This behavior can be observed in Figure 5 with a variation of the hot water supply temperature from 25 to 55 °C. The shape of the curves respects the usual behavior of the effect of the hot water supply temperature on the permeate flux studied in the literature [9,15]. The exponential increase of the permeate flux observed can be explained by the increase of the vapor pressure (the actual driving force) with temperature according to Antoine’s law. The results obtained with the module are encouraging regarding the behavior. It can therefore be concluded that the effect of the coolant temperature on the permeate flux is less significant than the effect of the hot water supply temperature. The preliminary results also validate the design of the AGMD module.

### 2.3. Description of the Experimental Coupling Setup

**Figure 6:**
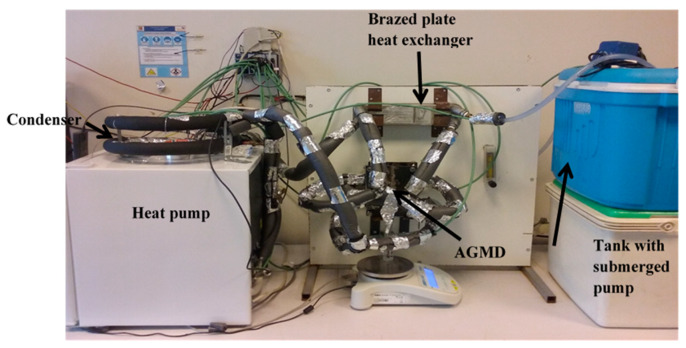
Photograph of the heat pump prototype coupled to the AGMD unit.

The laboratory scale experimental installation (Figure 6) includes, on the one hand, the prototype heat pump and, on the other hand, an AGMD module, a storage tank, and a heat recovery exchanger. The tubular exchanger, the heat pump condenser, transfers thermal energy from the refrigerant to the water without mixing them. The panel between the heat pump and the water tank supports the AGMD module and at the top, the recovery exchanger. The various elements are arranged in order to reduce the length of the water circuit and to minimize hydraulic pressure drops and thermal losses. The same instrumentation as for the preliminary tests was used for the coupled prototype.

**Figure 7:**
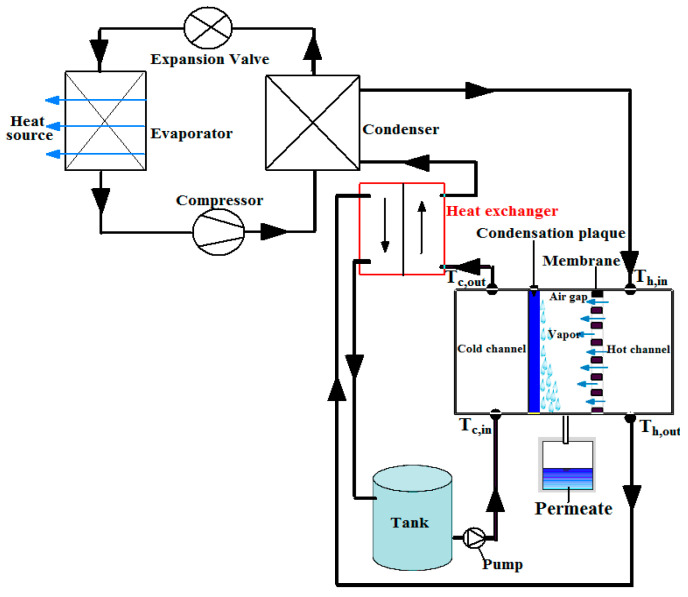
Scheme of the heat pump prototype coupled to the AGMD unit. Reprinted from [9] with permission from Elsevier.

On this bench, two circuits exist: a water circuit (hydraulic circuit) and a refrigerant circuit. The cooling water circuit and that of the hot feed are coupled in this experimental study. Only one pump is therefore necessary in the hydraulic network. This single circuit is only possible with AGMD and PGMD (permeate gap membrane distillation) and not with DCMD, VMD, and SGMD which require an auxiliary pump. This is one of the reasons for choosing AGMD for coupling with a heat pump. The hydraulic circuit contains no water cooling device of the cold channel. The vapors in the air gap of the AGMD module are condensed by heat exchange with water at room temperature from the tank. The condensates are collected in a test tube placed on the scales. The scheme of the prototype is shown in Figure 7. It is taken from the previously published design study [9]. In this system, the hot feed flow rate is the same as the cold flow rate. Reynolds numbers in the feed compartment of the membrane module are in the laminar range (6 to 28 depending on the tests) for all the tested operating conditions.

#### 2.3.1. Refrigerant Circuit

The choice of a very small refrigerator was made for cost reasons and in order to easily control the ambiance around the experimental set-up. The refrigerant circuit operates with isobutene R600a, which is not the most used in cooling devices. However, the operating temperatures of the sources are negligibly affected by the refrigerant choice. Moreover, the coefficient of performance is very close in nominal operating conditions between R600a and the most commonly used refrigerant R134a [49]. It essentially comprises an evaporator, a compressor, a water condenser (coaxial tube-in-tube exchanger), and a capillary tube as expansion device. The evaporator device is located inside the refrigerator. R600a at low temperature in the evaporator recovers heat from the air contained in the refrigerator cabinet. The refrigerant heats the water of the hydraulic circuit through the condensation energy.

#### 2.3.2. Hydraulic Circuit

The water circulation in the hydraulic circuit is ensured by a small 5 W aquarium pump placed in the water tank. Under nominal operating conditions (i.e., energetically optimal operating conditions), it has a pumping height of 0.7 m and produces a flow rate of 370 L/h. In this study, the flow rate considered were in the range 0–4 L/h. The water first passes through a flowmeter (0.5 to 5 L/h) and a valve adjusts the flow in the hydraulic circuit to supply the cold channel of the AGMD unit. The output of the latter is connected to a brazed plate heat exchanger (BPHX). Water is passed to the coaxial exchanger as it exits the plate exchanger. The BPHX is composed of two tubes wound in a spiral, the refrigerant circulates in the central tube while the water circulates in counter-current in the annular space between the inner and outer tubes. The water is brought to high temperature and then passes into the hot channel of the AGMD cell. The thermal energy contained in the brine is recovered by the BPHX and enables to preheat the water entering the condenser and slow down the temperature progression of the feed solution in the tank.

### 2.4. Performance Analysis

In this study, the performance of the coupling of the heat pump and the MD unit was evaluated in terms of four indicators: the permeate flux (PF), the gained output ratio (GOR), the coefficient of performance (COP), specific thermal energy consumption (STEC), and the specific electrical energy consumption (SEEC).

The permeate flux is defined as follows:

(1) $$\mathrm{PF}=\frac{\Delta m}{\Delta t\cdot A_{m}}$$

with Δm is the permeate mass collected (kg), Δt is the sampling time (h) and A_m_ is the effective membrane surface (m^2^).

For desalination units, the energetic performance is evaluated by the amount of energy consumed with respect to the amount of fresh water produced. The GOR corresponds to the ratio of the amount of energy necessary to vaporize the permeate flux divided by the heat consumption. It is one of the indicators generally used to assess the energy efficiency of thermal desalination systems. The GOR is used to measure the energy consumption of the process and it can be described as follows:

(2) $$\mathrm{GOR}=\frac{\mathrm{PF}\cdot \rho_{p}\cdot A_{m}\cdot \Delta h_{v}}{{\dot{Q}}_{\mathrm{cd}}}$$

where $\Delta h_{v}$ is the latent heat of vaporization and ${\dot{Q}}_{\mathrm{cd}}$ is the heating capacity of the heat pump defined as:

(3) $${\dot{Q}}_{\mathrm{cd}}=\mathrm{FFR}\cdot \rho_{f}\cdot {\mathrm{cp}}_{f}\cdot \left(T_{h,\mathrm{in}}-T_{h,\mathrm{out}}\right)$$

with $\rho_{p}$ and $\rho_{f}$, respectively, the permeate and feed density, FFR is the feed flow rate (hot channel), ${\mathrm{cp}}_{f}$ is the feed specific heat capacity and $T_{h,\mathrm{in}}$ and $T_{h,\mathrm{out}}$ are, respectively, the temperatures in and out of the hot channel.

In these studies, the compressor consumption and the COP are evaluated. The COP is defined as the ratio of the cooling power ${\dot{Q}}_{\mathrm{ev}}$ to the electrical power absorbed by the compressor ${\dot{W}}_{\mathrm{elec}}$ (Equation (4)).

(4) $$\mathrm{COP}=\frac{{\dot{Q}}_{\mathrm{ev}}}{{\dot{W}}_{\mathrm{elec}}}$$

The cooling capacity ${\dot{Q}}_{\mathrm{ev}}$ is determined from an energy balance applied to the refrigerator Equation (5):

(5) $${\dot{Q}}_{\mathrm{ev}}+{\dot{W}}_{\mathrm{elec}}={\dot{Q}}_{\mathrm{cd}}$$

Another performance indicator that can be used to assess the energy efficiency of the systems is the STEC. It corresponds to a measurement of the thermal energy consumed by the system to produce one cubic meter of fresh water (kWh/m^3^). It is an important parameter characterizing the performance of a desalination process, in particular from the point of view of the overall durability of the process [50]. The STEC is given by the following equation:

(6) $$\mathrm{STEC}=\frac{{\dot{m}}_{f}\cdot {\mathrm{cp}}_{f}\cdot \left(T_{h,\mathrm{in}}-T_{h,\mathrm{out}}\right)}{\mathrm{PF}\cdot A_{m}}$$

The specific electrical energy consumption (SEEC), which reports the amount of electrical energy required to produce one cubic meter of distillate water, is often neglected in studies examining MD systems [51]. For the coupling of a heat pump and a membrane distillation unit, it is important to study the SEEC. The specific electric energy consumptions of the coupling system and the part of the MD are determined as follows:

(7) $$\mathrm{SEEC}\_{\mathrm{HPMD}}_{}=\frac{{\dot{W}}_{\mathrm{elec}}}{{\mathrm{PF}\;A}_{m}}$$

(8) $$\mathrm{SEEC}\_{\mathrm{MD}}_{}=\frac{{\dot{W}}_{\mathrm{elec}}-{\dot{W}}_{\mathrm{Con}}}{\mathrm{PF}\;\cdot A_{m}}$$

where ${\dot{W}}_{\mathrm{Con}}$ presents the rate of electrical energy consumption by the refrigerator only in normal operation, this is given by the manufacturer.

## 3. Results and Discussion

The performance of the coupled system is calculated from transient and stationary test records to observe the behavior of the experimental setup. The compressor “continuous” and “controlled” operations are the two modes used to study the performance of the prototype. The compressor continuous mode of operation corresponds to a configuration where the refrigerator door is permanently open. The compressor is forced to operate continuously because the set point for the indoor air temperature is never reached. This configuration simulates the use of the heat pump for air conditioning purpose. In controlled mode, the door of the refrigerator is closed and an on/off thermostat controls the ambient temperature inside the refrigerator mimicking a storage mode (e.g., food storage). The cooling and electrical power measured during the tests are almost stable and around 60 W and 50 W respectively. The estimated COP is equal to 1.2. The water in the tank is at the room temperature of 19 ± 1 °C for all tests performed.

### 3.1. Continuous Compressor Operation (Door Wide Open)

#### 3.1.1. Dynamic Evolution of High Pressure

**Figure 8:**
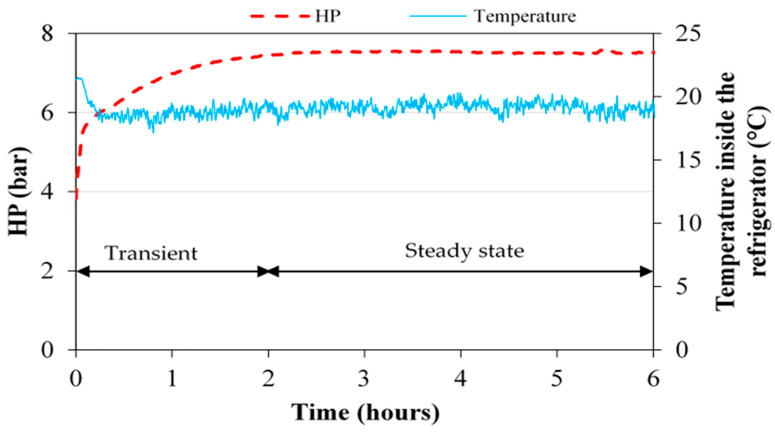
High pressure and the temperature of the air inside the refrigerator during a start-up test in continuous mode.

In the door wide open mode (continuous or air conditioning mode), the evolution of the refrigerant high pressure and the air temperature determine the time required for the refrigeration cycle to operate at a stable high pressure with the feed flow rate of 1 L/h (Figure 8). This pressure depends slightly on the temperature of the air inside the refrigerator. The stabilized pressure is obtained after 2 h of operation. Before stabilization, the heat fluxes from the moving parts of the compressor towards the refrigerant and the ambiance are not balanced. In the following results, the mean values will be calculated without the transient phase.

#### 3.1.2. Effect of Flow Rate on Hot Channel Inlet Temperature and Permeate Flux in the Continuous Mode

The influence of the feed flow rate on the coupled unit has been investigated in the range from 1 to 4 L/h.

**Figure 9:**
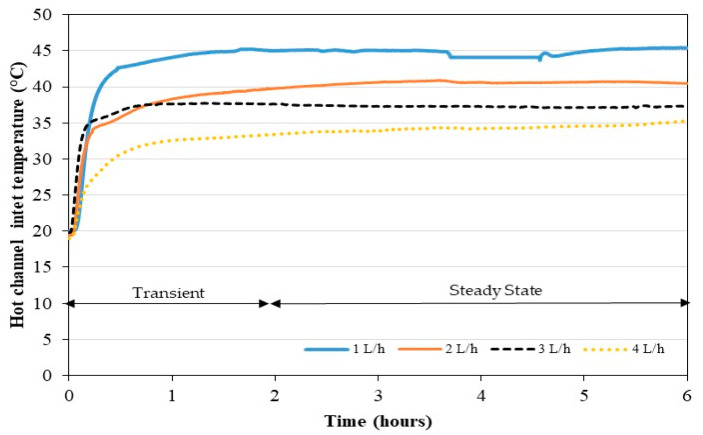
Effect of the flow rate on the water inlet temperature in the hot channel in continuous mode.

**Figure 10:**
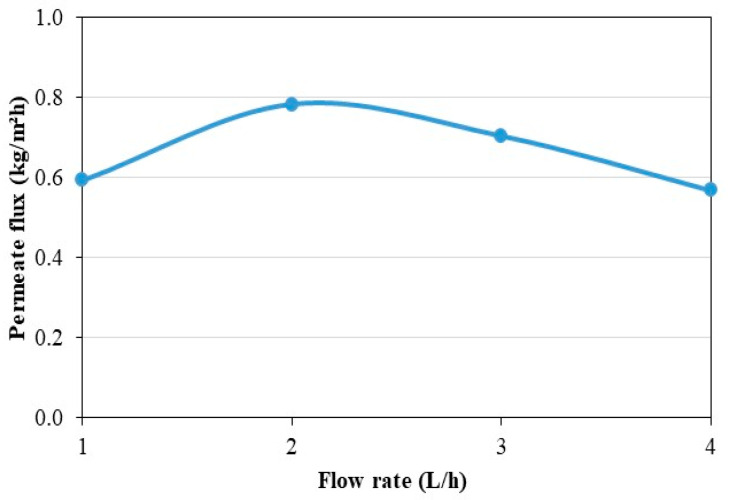
Effect of flow rate on permeate flux in continuous mode.

Figure 9 illustrates the temporal evolution of the inlet temperature of hot water in the distillation unit for the different feed flow rates considered. As the thermal power does not vary much, the water outlet temperature from the condenser (being the same as the MD unit inlet temperature) increases as the feed flow rate decreases. The BPHX preheats the feed solution by about 5 K when the steady state is established and cools the brine before it returns to the feed tank. This effect is not visible in Figure 10. A relative stability of temperatures is obtained after 2 h, which is in accordance with the stability of high pressure that can be observed in Figure 8. In addition, the residence time of the feed water in the condenser is longer when the flow is low. Accordingly, a low flow rate is in favor of a high-water inlet temperature in the hot channel.

Figure 10 shows the values of the permeate flux for the different feed flow rates during the 4 h of the steady state. The average temperatures at the steady state in the tank are at the room temperature around 20 °C and increase by 5 K during the period. For each experiment, the feed water temperature is the one displayed on Figure 10. Parametric studies from the literature show that feed fluid temperature and flow rates have a significant effect on the MD permeate flux [52,53]. As shown in Figure 9, a direct interplay exists between these two parameters due to the coupling of the heat pump and the MD unit. Therefore, when the feed flow rate increases the feed temperature at the entrance of the MD module decreases. Such complex behavior can complicate the analysis of the permeate flux results. Working with the largest feed flow rate does not lead to the highest permeate flux, which would not have been the case with a simple MD module (without coupling with a heat pump). This can be explained by the fact that, if the feed flow rate increases, the convective heat exchange coefficient increases in the distillation unit but the feed water temperature decreases. The permeate flux being lower at higher flow rate suggests that the temperature decrease has more effect on MD performance than the convective heat exchange increase. In addition, it can be observed in Figure 10 that the use of a flow rate of 2 L/h is more advantageous for the permeate flux compared to other flows rates studied. Under these operating conditions, there is an optimal flow rate to produce fresh water.

**Figure 11:**
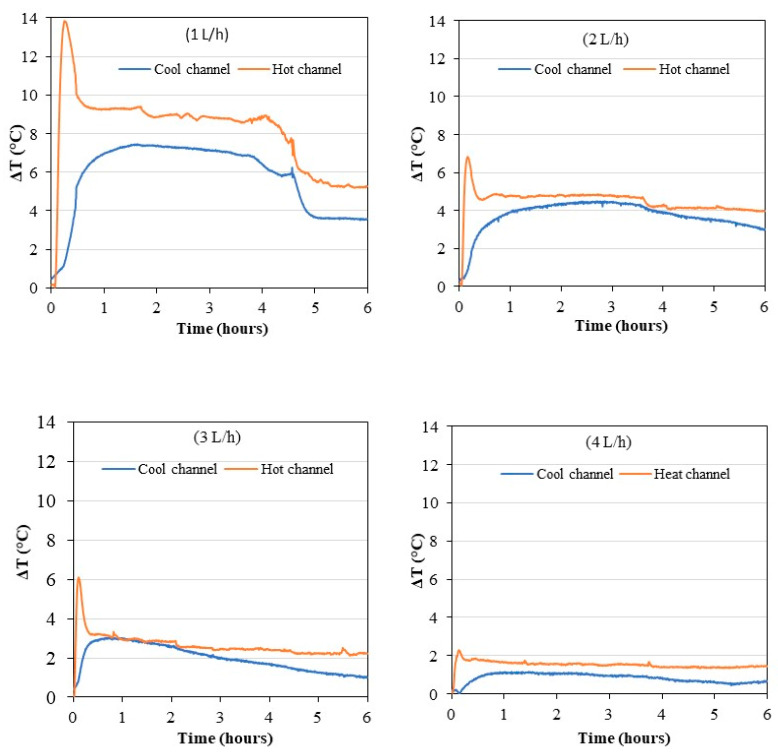
Evolution of the temperature differences of the channels at the different operating flow rates in continuous mode.

To understand the behavior of the permeate flux with respect to the variation of the feed flow rate, the temperature differences between the inlets and outlets of the AGMD unit channels are shown in Figure 11. The temperature increases in the cold channel and decreases in the hot channel. In general, the temperature differences both in the hot and cold channels are greater when the flow rate is lower. However, with flow rates of 2 and 3 L/h, the difference in average channel temperatures is almost halved compared to that with a flow of 1 L/h.

Large temperature differences in the cell can increase the share of parasitic exchanges, through the membrane module elements, between the cold compartment and the hot compartment of the cell. The membrane and the casing of the unit itself can also act as a heat exchange medium between the top and bottom of the cell. Finally, higher heat fluxes by conduction can be generated through the membrane and the casing and become influential for low Reynolds numbers. The actual average temperature of the hot water at the interface with the membrane can be decreased and the actual average temperature of the cold channel can be increased. These phenomena can lead to a reduction of the difference in transmembrane pressure that decreases the permeate flux. Such behavior can also be linked to the geometry of the channels because the volume of the cold compartment is greater than that of the hot one. Heat losses seem to have more impact on the hot channel because the differences between inlet and outlet are generally smaller, and especially for the lowest flow rate. Indeed, the heat losses are proportional to the temperature difference between the water and the ambience, which is greater for the hot water. The lack of temperature homogeneity in the feed tank leads to important cold water temperature variation with time. This phenomenon indirectly impacts the hot water temperature. This effect is observed to be lower when the flow rate increases thanks to better mixing inside the tank.

#### 3.1.3. Energy Analysis in Continuous Mode

**Figure 12:**
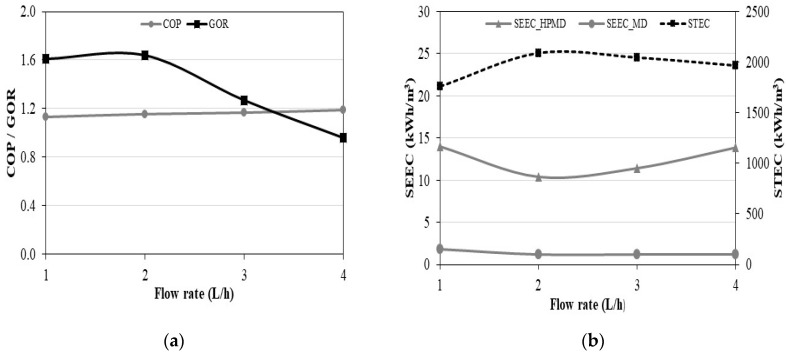
Effect of flow rate, on (**a**) COP and GOR, (**b**) STEC and SEEC in continuous mode.

For such a compact system whose scalable parameters are very close, it is very important to show whether the system is efficient or not. The COP characterizes the performance of the heat pump. GOR, STEC, SEEC_MD, and SEEC_HPMD are used to evaluate the performance of the coupled system. The effect of the flow rate on these indicators is presented in Figure 12. The COP shows a linear positive relationship with the feed water temperature, while the GOR, STEC, SEEC_MD, and SEEC_HPMD show a nonlinear relationship. The flow rate has a small effect on the COP. The GOR is higher at low flow rates (1 and 2 L/h) due to the high outlet water temperatures of the heat pump condenser favoring a decrease of the enthalpy of vaporization inside the MD unit (Figure 12a). At the flow rate of 2 L/h, STEC is higher and SEECs are lower (Figure 12b). Even if the thermal energy is higher, it is compensated by the higher GOR. The minimum SEEC values are obtained when the permeate flux is maximum, at the flow rate of 2 L/h. The SEEC_HPMD values are consistent with the results from the literature (4.5–70.2 kWh/m^3^) [50,53,54,55,56,57,58]. However, the SEEC_HPMD takes into account the energy consumed for the production of cooling energy, which is not done in other studies. Therefore, the SEEC_MD is used to make a reasonable comparison. It only takes into account the excess energy consumed by the compressor subtracting the electric power under nominal operation given by the manufacturer to the electric power measurement including the power consumption of the circulation pump. The results of SEEC_MD show that the heat pump for simultaneous cooling and desalination by membrane distillation is more efficient than other systems from the literature.

### 3.2. Controlled Compressor Operation (Door Is Closed)

This mode of operation corresponds to a normal configuration where the refrigerator door is closed. An on/off thermostat controls the inside air temperature of the heat pump prototype (controlled mode or food storage mode). The upper and lower boundaries of the controller are 0 and 3 °C.

#### 3.2.1. Evolution of the Air Temperature inside the Refrigerator Cabinet

**Figure 13:**
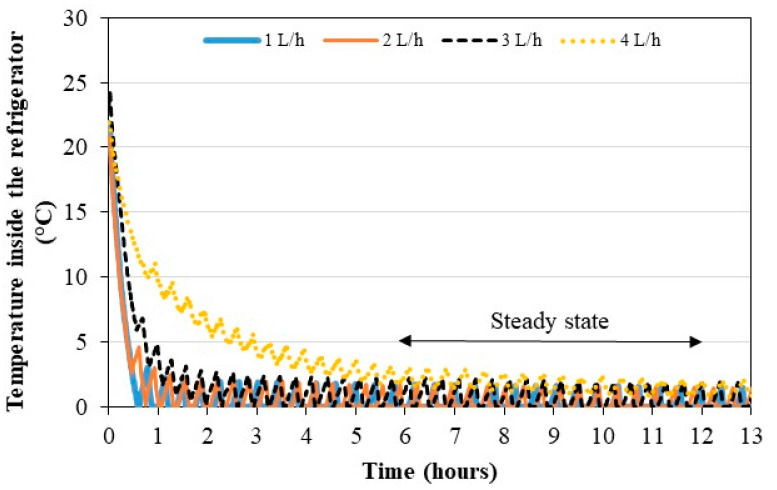
Dynamic behavior of the refrigerator air temperatures for different operating flow rates in controlled mode.

The same operating flow rates were used to study the dynamic behavior of the indoor air temperature inside the refrigerator cabinet (Figure 13). In less than 1 h of operation, the heat pump prototype manages to reduce the air temperature of the refrigerator by about 20 °C compared to the initial temperature with the flow rates of 1, 2 and 3 L/h. A transient phase of 1 h is observed after turning on the compressor. For a flow rate of 4 L/h, a transient phase of more than 4 h is observed. The lower condensing pressure causes a low pressure decrease and the refrigerant evaporates at a lower temperature inside the evaporator. The cooling capacity is also lower. Therefore, the difference between the temperature of the internal air of the refrigerator and the evaporation temperature of the refrigerant increases. The temperature sensor of the controller will stop the compressor more quickly, before the measured temperature is 0 °C. Beyond these transient phases, the regime is established with temperatures varying from 0 to 3 °C, according to the upper and lower boundaries of the on/off controller. In controlled mode (door fully closed), the pseudo-steady-state is established for all flow rates between 6 and 12 h of operation.

#### 3.2.2. Effect of the Flow Rate on the Hot Channel Inlet Temperature and the Permeate Flux in Controlled Mode

**Figure 14:**
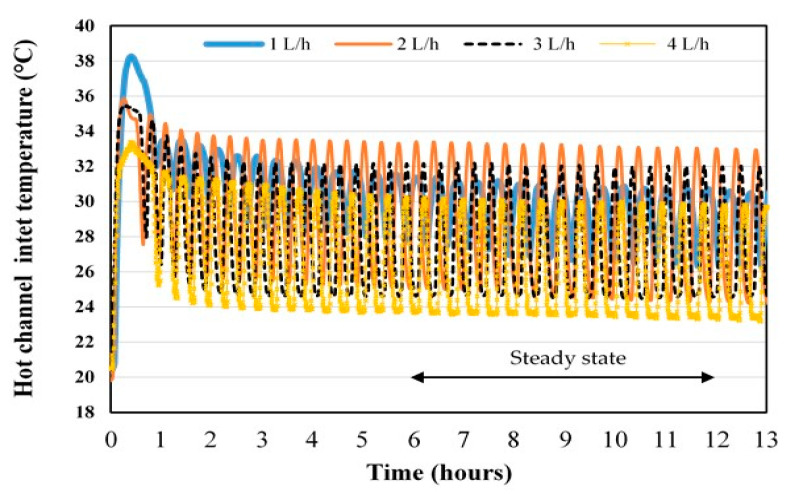
Evolution of the inlet temperatures of the hot channel in controlled mode.

The variation of the inlet temperature of the hot channel is shown in Figure 14. When the compressor stops working, it is observed that the temperature is lower with the flow rate of 4 L/h and when the compressor is running that it is at a maximum with the flow rate of 2 L/h. For a better analysis of Figure 14, the average inlet temperatures of the hot channel and the average differences between temperature maxima and minima over the pseudo-steady-state period are presented in Table 3. This table shows that the average hot channel inlet temperature decreases with increasing flow rate. The same effect of flow rate on the hot channel inlet temperature was observed for the air conditioning mode. The maximum temperature difference is obtained with the flow rate of 2 L/h. The low evolution of the tank temperature was also checked with less than 1 K difference for the flow rates tested.

**Figure 15:**
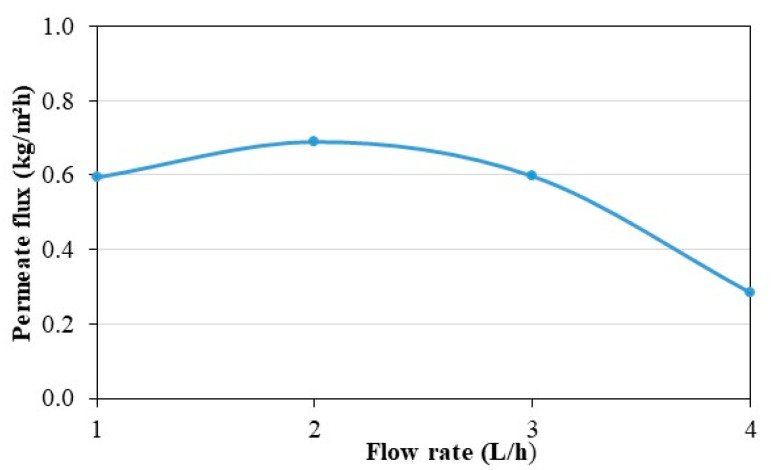
Effect of flow rate on permeate flux in controlled mode.

The permeate flux depending on the feed flow rate is shown in Figure 15. Interestingly, it was previously observed in Figure 13 that the temperature inside the refrigerator was the same at steady state whatever the feed flow rate. In this configuration, an increased water production (higher permeate flux) does not affect the temperature of the air inside the refrigerator compared to the operating conditions leading to the lower water production. In general, the permeate fluxes are lower in Figure 15 than in Figure 10 because the hot water temperature is lower. In Figure 15, the feed flow rate of 2 L/h has been observed to be the highest according to the permeate flux optimization. In the pseudo-steady-state zone, the cumulative time of operation of the compressor is longer for 1 L/h and shorter for 2 L/h. This can be justified by the fact that heat losses are greater for 1 L/h (Figure 11).

**Figure 16:**
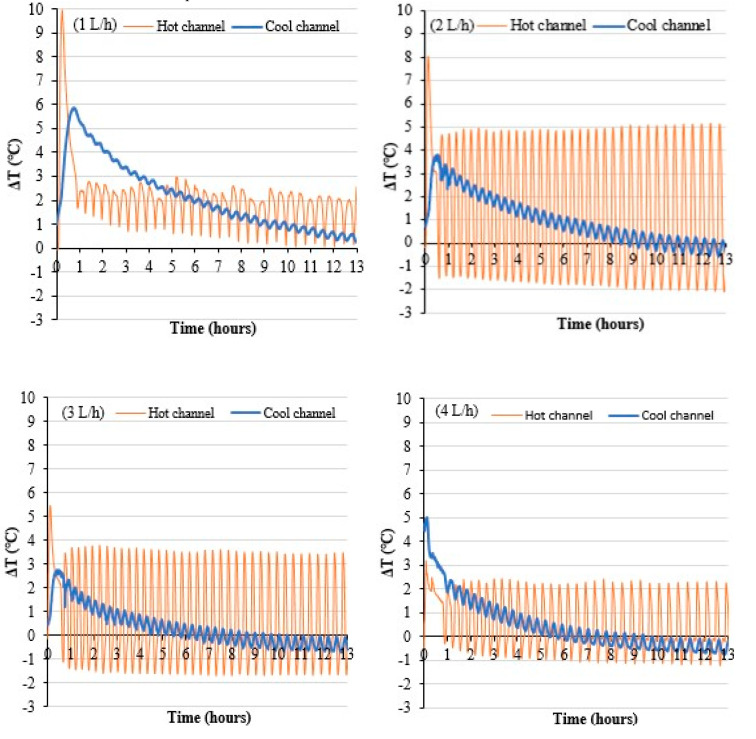
Evolution of channel temperature differences in controlled mode.

The on/off control of the compressor in storage mode gives a particular dynamic to the temperatures of the hot and cold fluids in the AGMD module as seen in Figure 16. This figure shows the temperature differences of water entering and leaving the MD unit. This behavior is partly due to the compressor on/off operating periods, which differ depending on the flow rate. The period is longer when the flow is low. It happens at some points that the outlet temperature of the condenser is lower than the cooling temperature of the cold channel. Such behavior is due to the cooling of the water in the tube-in-tube condenser when the heat pump stops working. The temperature amplitudes are smaller with increasing flow, except with 1 L/h. This study also concludes to the existence of an optimal flow rate around 2 L/h under these experimental conditions for our prototype. The coupling of the heat pump and the desalination unit lead to an optimum for both configurations dependent on heat exchange coefficients and heat losses linked to high temperature.

#### 3.2.3. Energy Analysis in Controlled Mode

**Figure 17:**
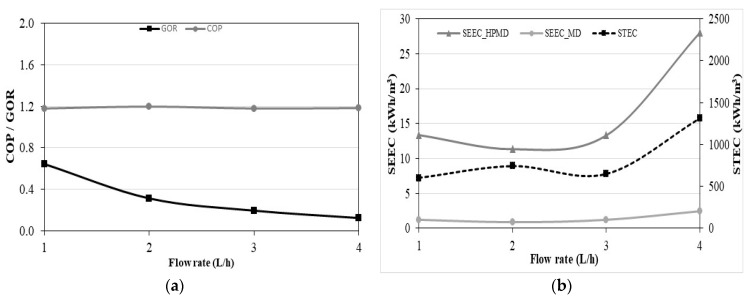
Effect of flow rate, on (**a**) COP and GOR, (**b**) STEC and SEEC in food storage mode.

Figure 17 illustrates the effect of the flow rate of energy performance indicators in the controlled mode. The variation of the flow rate has an even lower effect on the COP (Figure 17a) than in the continuous mode. An increase of the flow rate significantly decreases the permeate flux and the GOR. Figure 17b shows that the increase of the flow rate does not influence STEC and SEEC for 1, 2 and 3 L/h. For the flow rate of 4 L/h, the STEC and the SEEC increase drastically. This behavior is due to the drop of the permeate flux observable in Figure 15. The average STEC in the controlled mode is 824.75 kWh/m^3^, which is 2.4 times lower than the average STEC value in the continuous mode. This shows heat recovery is more important than with continuous mode than with food storage. The minimum SEEC_HPMD value of 11.33 kWh/m^3^ was obtained at the flow rate of 2 L/h. This value is slightly higher than the smallest value obtained with the continuous mode 10.38 kWh/m^3^. The lowest value of the SEEC_MD is 0.88 kWh/m^3^ compared to 1.17 kWh/m^3^ for the continuous mode.

## 4. Conclusions

This article presents the prototype of heat pump for simultaneous cooling and desalination by membrane distillation. The results obtained and presented in this study clearly demonstrate the potential of an AGMD unit to be coupled with a heat pump, MD being able to work at the low temperature levels encountered at the heat pump condenser.

The effect of feed water flow rate on various indicators such as permeate flux, GOR, cold production, COP, STEC, and SEEC were investigated and are presented in this paper. The necessity to study and optimize directly the coupled system is demonstrated instead of studying the heat pump and the MD system separately. Therefore, in AGMD, it has been shown in the literature that the productivity (permeate flux) increases with the feed flow rate. However, for the case of coupling the heat pump with the AGMD, the higher feed flow rate does not increase the amount of distilled water. A higher flow rate enhances the convective heat transfer coefficient but reduces the outlet hot water temperature. An optimal flow rate of 2 L/h was found for the prototype presented. The heat pump prototype has limitations linked to its small dimensions in terms of high heat losses which directly impact the energetic performance of the system. The hydrodynamic of the membrane module can also be improved.

The experimental results obtained with this prototype will allow us to validate a numerical model of the coupled system. The model will be used to simulate other CO_2_ and R290 heat pumps because these refrigerants have interesting thermodynamic and environmental properties. Furthermore, the model will be corrected and used to simulate the performance of the coupled heat pump/AGMD system at the building scale. Thus, it will be of high importance to be able to evaluate the scale-effect on the performance and costs of the proposed technology while taking into account other structural parameters such has building location, seasonality, weather variation, etc.

The advantage of the coupling of the heat pump with the MD process is to valorize the thermal heat rejected by the refrigeration equipment to produce water. This new design can also be coupled with renewable energies (in particular solar photovoltaic systems or wind turbines) in order to build standalone off-grid systems.

To conclude, this work contributes to the innovation effort in desalination processes by membrane distillation especially regarding the reduction of energy consumption, which has ever been a major concern for desalination systems. This system is aimed at hotels, offices, naval applications, and unconnected remote locations. However, it has to be kept in mind that the driver for the implementation of such systems should be the need for refrigeration (and not only freshwater) as this system might not be competitive for freshwater production alone (as shown by the elevated STEC values found in this study). Therefore, using this technology, freshwater production should be envisaged as an added value to refrigeration systems.

## Figures and Tables

**Table 1:** Comparison of different membrane distillation techniques [17].

MD Technique	Acronym	Advantages	Drawbacks
Direct contact	DCMD	Simplicity	Higher wettability
Air gap	AGMD	Simplicity, Low wettability	Lower permeate flux
Sweeping gas	SGMD	High permeate flux	External water condensation (additional device)
Vacuum	VMD	High permeate flux	External water condensation (additional device)
Permeate gap	PGMD	Simplicity	Higher wettability

**Table 2:** Measurement methods and uncertainties.

Value	Sensor/Measurement Method	Uncertainties
Pressure sensors	Johnson Controls	±1%
Temperature	Type K Thermocouple	±0.5 °C
Precision scales	Adam Nimbus	0.01 g
Flow rate	Variable area flowmeter	10%
Power	Current transformer	±1%

**Table 3:** Average hot feed temperature and temperature difference between on/off in pseudo-steady state.

Flow Rate (L/h)	Average Hot Feed Temperature (°C)	Average Temperature Difference (K)
1	30.29	4.20
2	29.3	7.00
3	28.08	6.60
4	26.90	6.40

## Data Availability

The data are not publicly available for confidentiality reasons.

## Abbreviations

AGMD	air gap membrane distillation
DCMD	direct contact membrane distillation
HP	heat pump
HPMD	heat pump and membrane distillation
MD	membrane distillation
PLA	polylactic acid
PGMD	permeate gap membrane distillation
SGMD	sweeping gas membrane distillation
VMD	vacuum membrane distillation
**Variables:**	
cp	Specific heat capacity (kJ/kg K)
COP	Coefficient of performance (-)
Δ	difference
ρ	Density (kg/m^3^)
FFR	Feed flow rate (m^3^/s)
GOR	Gained output ratio (-)
h	Enthalpy (kJ/kg)
m	mass (kg)
PF	Permeate flux (kg/m²h)
SEEC	specific electric energy consumption (kWh/m^3^)
STEC	specific thermal energy consumption (kWh/m^3^)
T	temperature (K)
t	time (h)
$\dot{W}$	mechanical power (W)
**Subscripts:**	
c	cold
cd	condenser
elec	electric
ev	evaporator
f	feed
h	hot
in	inlet
nom	nominal
out	oulet
p	permeate
v	vaporization
